# On Intoxication

**Published:** 1848-09

**Authors:** 


*On Intoxication.*—By G. CORFE, Esq., Middlesex Hospital.

[Mr. Corfe has lately given us a series of very practical articles on different subjects, many of which we shall place before our readers in due course. The first we give is "on Intoxication," to distinguish which, from cerebral diseases, is sometimes puzzling. Mr, Corfe says—]

A patient is brought into the hospital, perhaps on a policeman's stretcher, or he is carried in by friends, who state that he was picked up in the streets, senseless. His pupils are dilated, immovable; his breathing is deep and low, and heavy, the expiration being short and abrupt, whilst the inspiration is a prolonged, deep sigh, with more or less stertor: pulse is full and strong. The suspicion arises that the man has sanguineous effusion into one or both ventricles of the brain. But it must be again acknowledged that, of all the perplexing, deceitful, and varying symptoms which diseases occasionally put on, those of cerebral lesions or more cerebral disturbance are, of all others, the most difficult to decide upon. We have admitted cases into the hospital in the dead of night, brought here by policemen, who have found the patient lying senseless, or he has been seen to fall senseless on the pavement: we have bled, blistered, leeched, and purged; shaved the head, and given turpentine enemas, but all to no purpose; insensibility has remained; when, to onr surprise, in twelve hours afterwards we have gone to visit our patient we have found him perfectly sensible and tolerably well, not more surprised at the loss of a head of hair, than we have been at the sudden revival of our supposed case of appoplexy. Whilst on the other hand, I have admitted a case as one of "dead drunk," perfectly inanimate, and have, for the sake of precaution, sent him into the ward to bed, and yet have found it to prove an instance of apoplexy. I have, however, learned a valuable lesson by even these difficulties: for in every instance of late years, when a case of complete insensibility is admitted, I have requested the house-surgeon to empty, and then wash out the stomach by means of the pump; and, if it has been from drunkenness, the "sot" has shown his character up before we have finished this operation; and if it has been one of appoplexy, it has done no harm, and it has proved that it was more than intoxication, as no alcoholic fetor has been detected in the contents of the stomach.

In all cases of intoxication the mental faculties become roused before the operation of thoroughly washing out the stomach by the pump is concluded; whiist, on the other hand, it may be said, the reverse is ordinarily the case in cerebral lesions, or in mere concussions of the brain. Before we allow the tube to be withdrawn from the stomach, in cases of inebriety, we usually inject three ounces of the dilute acetate of ammonia draught, which has an extraordinary, effect sometimes, in "sobering" the head, and calming the stomach too.

I am aware that it is much easier to recommend the employment of the stomach-pump than to administer it to persons who are sometimes in the highest state of excitement bordering on maniacal fury. I am anxious, therefore, to describe the mode in which we employ this remedial agent in such cases, for until I arrived at this practical method, we were often obliged to abandon the use of the pump, in consequence of the difficulties which the struggles and powerful action of the patient's body produced; and I may here remark that, on one occasion, the gag was forced from the mouth, and the man bit the tube in twain a few inches from the cylinder, leaving the remainder, nearly two feet in length, in his œsophagus and stomach, when, by the greatest manual force, I wrenched open his jaws and plunged my fingers down his throat, and was fortunate enough to seize the divided end at the point of finger and thumb just as it had slipped within the bag of pharynx. Since that period I adopted the following method:—The patient is seated in a strong wooden chair, another chair is placed behind him, and an attendant is ordered to sit in it, and taking the arms of the patient, he pinions them by holding the wrists firmly against the back of the chair. This method serves to fasten the trunk securely in the chair; the legs are then swung in a round towel, which is passed round the ankes by a noose, and a second chair being placed so that the back of it shall be towards the legs of the patient, another attendant is placed in it; he carries the towel over the back of the chair, and sits upon it, and thus the legs are at right angles with the trunk, and consequently they are almost powerless. If, however, the man offers to flex the knees, the ankles are instantly raised higher, and the power of the flexors of the thigh is thereby overcome in an instant. By this position, it will be observed, that the patient is deprived of all muscular power, and the only fixed point on which his body rests is the ischiatic tubera. I am satisfied that the stomach-tube may not only be introduced with comparative ease, but that the operation of the pump is perfectly harmless, when judiciously administered, upon a refractory patient thus immovably fixed.—*Medical Times, Oct.* 23. 1847, *p.* 17.—*Braithwaite's Retrospect.*

